# Bridging Targeting Precision and Oncologic Safety: Localization Accuracy for Margin Adequacy in Cone-Beam Computed Tomography-Guided Pulmonary Nodule Resection

**DOI:** 10.3390/cancers18142356

**Published:** 2026-07-21

**Authors:** Yu-Hsiang Wang, Hsu-Chih Huang, Chih-Yi Chen, Jiun-Yi Hsia, Guo-Zhi Wang, Ming-Chih Chou, Frank Cheau-Feng Lin

**Affiliations:** 1 Division of Thoracic Surgery, Chung Shan Medical University Hospital, Taichung 402, Taiwan; 2 Institute of Medicine, Chung Shan Medical University, Taichung 402, Taiwan; 3 School of Medicine, Chung Shan Medical University, Taichung 402, Taiwan

**Keywords:** pulmonary nodules, thoracoscopic surgery, localization accuracy, pathological margin, spherical error probable, wedge resection

## Abstract

**Background/Objectives:** Ground-glass pulmonary nodules are often nonpalpable and not visible during thoracoscopic surgery, making accurate localization important for achieving adequate pathological margins. However, no clinically validated localization-error threshold has been established. We evaluated the association between Dmn and pathological margin adequacy after image-guided thoracoscopic pulmonary nodule resection and sought to identify a clinically relevant localization-accuracy threshold. **Methods:** We retrospectively reviewed 169 patients in a predefined peripheral-lesion cohort who underwent cone-beam computed tomography-guided pulmonary nodule localization using hook-wire placement or dye marking in a hybrid operating room, followed by thoracoscopic wedge resection. Dmn was defined as the shortest three-dimensional Euclidean distance from the localization needle tip to the tumor margin. Pathological margin adequacy was defined as a resection margin equal to or greater than the maximum tumor diameter. Logistic regression was used to identify predictors of pathological margin inadequacy, and receiver operating characteristic analysis was performed to determine the optimal Dmn cutoff. **Results:** Dmn was independently associated with pathological margin inadequacy (odds ratio, 1.617; 95% confidence interval, 1.356–1.928; *p* < 0.001). Receiver operating characteristic analysis yielded an area under the curve of 0.827 and an optimal Dmn cutoff of 4.90 mm, with a sensitivity of 71.0% and specificity of 94.2%. Pathological margin inadequacy occurred more frequently in patients with Dmn ≥ 4.9 mm than in those with Dmn < 4.9 mm (73.3% vs. 6.5%, *p* < 0.001). **Conclusions:** In this single-center cohort, a Dmn of approximately 4.9 mm was associated with pathological margin inadequacy after cone-beam computed tomography-guided pulmonary nodule localization and thoracoscopic wedge resection. This value may serve as a candidate intraoperative warning threshold for margin reassessment; however, prospective external validation is required before routine clinical implementation.

## Simple Summary

Ground-glass pulmonary nodules are often difficult to visualize or palpate during thoracoscopic surgery, making accurate localization essential for achieving adequate surgical margins. However, the degree of localization error beyond which margin adequacy becomes compromised remains unclear. In this retrospective study of patients undergoing cone-beam computed tomography-guided pulmonary nodule localization in a hybrid operating room, we evaluated whether Dmn, defined as the shortest Euclidean distance from the localization needle tip to the tumor margin, predicted pathological margin inadequacy after thoracoscopic wedge resection. A Dmn of approximately 4.9 mm was associated with a marked increase in margin inadequacy. This value may serve as a candidate intraoperative warning threshold prompting margin reassessment or refinement of resection planning. Prospective external validation is required before routine clinical use.

## 1. Introduction

Low-dose computed tomography screening has increased the detection of small, early-stage lung cancers. The TALENT (Taiwan Lung Cancer Screening in Never-Smoker) study reported a lung cancer detection rate of 2.6%, with 96.5% of detected cancers diagnosed at stage 0 or I [1]. Similar findings from screening programs in China and Korea indicate a broader regional trend toward the detection of subcentimeter pulmonary nodules [2,3]. Consequently, thoracic surgeons increasingly encounter small pulmonary nodules, particularly ground-glass nodules, that may require surgical diagnosis or treatment.

However, small or deep pulmonary nodules may be difficult to visualize or palpate during video-assisted thoracoscopic surgery (VATS). Suzuki et al. [4] reported a 54% conversion rate to thoracotomy, most commonly because the target nodule could not be identified, as well as a 63% probability of detection failure for nodules located more than 5 mm from the pleural surface. Image-guided localization has therefore become an important adjunct for small, deep, or ground-glass pulmonary nodules that are difficult to identify intraoperatively [5,6,7,8,9,10]. Hybrid operating rooms (HORs) further allow localization and resection to be completed during the same anesthetic session, thereby improving procedural coordination and workflow efficiency [11,12]. With optimized HOR protocols, more than 80% of multiple pulmonary nodules can be localized within 5 mm of the intended target [13,14].

Nevertheless, technical targeting precision alone does not establish the oncological adequacy of resection. In thoracoscopic sublobar resection, a more clinically meaningful endpoint is whether localization facilitates an adequate pathological margin, because inadequate margins, staple-line abnormalities, and local recurrence remain important concerns [15,16,17,18]. Previous studies of image-guided localization have primarily focused on technical success, complication rates, or targeting error, whereas the quantitative relationship between localization error and pathological margin adequacy remains insufficiently defined. Moreover, no clinically validated threshold has established the magnitude of localization error beyond which pathological margin adequacy becomes substantially compromised. Therefore, this study evaluated the association between three-dimensional localization error and pathological margin adequacy. Localization error was expressed as Dmn, defined as the shortest Euclidean distance from the localization needle tip to the tumor margin. We hypothesized that a greater Dmn would be independently associated with a higher risk of pathological margin inadequacy and that a clinically meaningful Dmn threshold could be identified. Accordingly, we sought to identify a candidate localization-accuracy threshold that could support intraoperative margin reassessment and localization-quality evaluation.

## 2. Materials and Methods

### 2.1. Patient Population

This retrospective study was conducted at a tertiary medical center and was approved by the Institutional Review Board of Chung Shan Medical University Hospital (approval No. CS1-24001; approval date: 12 April 2024). Patients who underwent localization of pulmonary ground-glass nodules (GGNs) in a hybrid operating room (HOR) between January and December 2022 were enrolled. The predefined primary analysis cohort comprised adults (≥18 years) with a single peripheral lung lesion, defined as a nodule located within the outer one-third of the distance from the hilum to the lung surface, consistent with the radiological definition used in the JCOG0802/WJOG4607L trial [19,20,21,22]. All patients had adequate pulmonary function and were classified as low surgical risk according to the American College of Chest Physicians’ guidelines [23].

At our institution, patients with persistent pulmonary GGNs underwent longitudinal assessment using serial thin-section CT. Surgical localization and resection were considered only when the lesion was judged to be highly suspicious for primary lung malignancy after multidisciplinary review of its interval radiological changes, morphology, solid component, and clinical characteristics. The term GGN was used as a radiological descriptor rather than as a specific etiological diagnosis.

**Figure 1:**
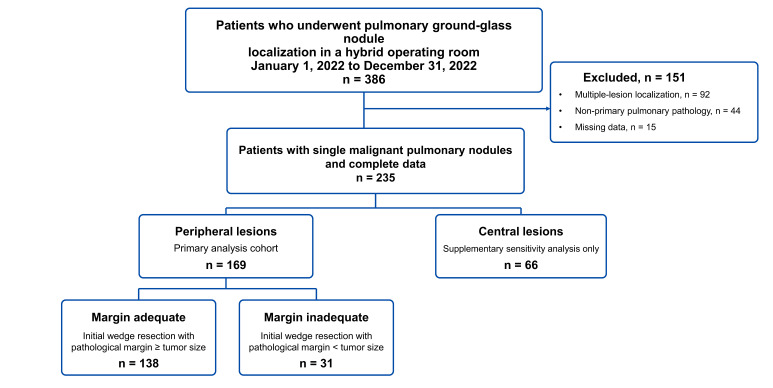
Study flow diagram. Patients who underwent intraoperative cone-beam computed tomography (CT)-guided pulmonary nodule localization in a hybrid operating room between January and December 2022 were screened. After exclusion of multiple-lesion localization, non-primary pulmonary pathology, and missing data, 235 patients with single malignant pulmonary nodules and complete data were identified. Among them, 169 patients with peripheral lesions, defined according to the JCOG outer one-third criterion, constituted the predefined primary analysis cohort and were stratified according to pathological margin adequacy. The remaining 66 patients with central lesions were not included in the primary analysis but were included in the supplementary sensitivity analysis. MA, margin adequate; MI, margin inadequate.

Inclusion criteria required malignancy on final pathology and complete data on tumor size, pathological margin, and localization coordinates. The final peripheral-lesion cohort consisted exclusively of pathologically confirmed primary pulmonary adenocarcinoma-spectrum lesions, including adenocarcinoma in situ, minimally invasive adenocarcinoma, and invasive adenocarcinoma. Patients who underwent multiple-lesion localization, had non-malignant final pathology or secondary pulmonary malignancy, or lacked essential data were excluded from the eligible cohort. Centrally located lesions were excluded from the predefined primary analysis but retained for the supplementary sensitivity analysis. The combined cohort of central and peripheral lesions included 235 patients. Patient enrollment, exclusions, and group stratification are summarized in Figure 1. The primary analysis cohort included 169 patients with peripheral lesions. The primary outcome was pathological margin adequacy (MA), defined as a pathological margin equal to or greater than the maximum tumor diameter [24,25,26,27]. Margin inadequacy (MI) was defined as a pathological margin smaller than the maximum tumor diameter. Because this retrospective cohort included all eligible patients treated during the study period, no a priori sample size calculation was performed. Therefore, the receiver operating characteristic (ROC)-derived cutoff was further assessed using bootstrap internal validation.

### 2.2. Localization Protocol and Surgery

Localization was performed under general anesthesia using a cone-beam CT system (C-arm CBCT; ARTIS Pheno^®^, Siemens Healthineers, Erlangen, Germany). Preoperative thin-section CT was used for longitudinal lesion assessment, radiological characterization, and procedural planning. After patient positioning and induction of general anesthesia, intraoperative CBCT was used to re-identify the target lesion, plan the needle trajectory using the integrated needle-guidance workstation, guide needle placement, and confirm the needle-tip position.

**Figure 2:**
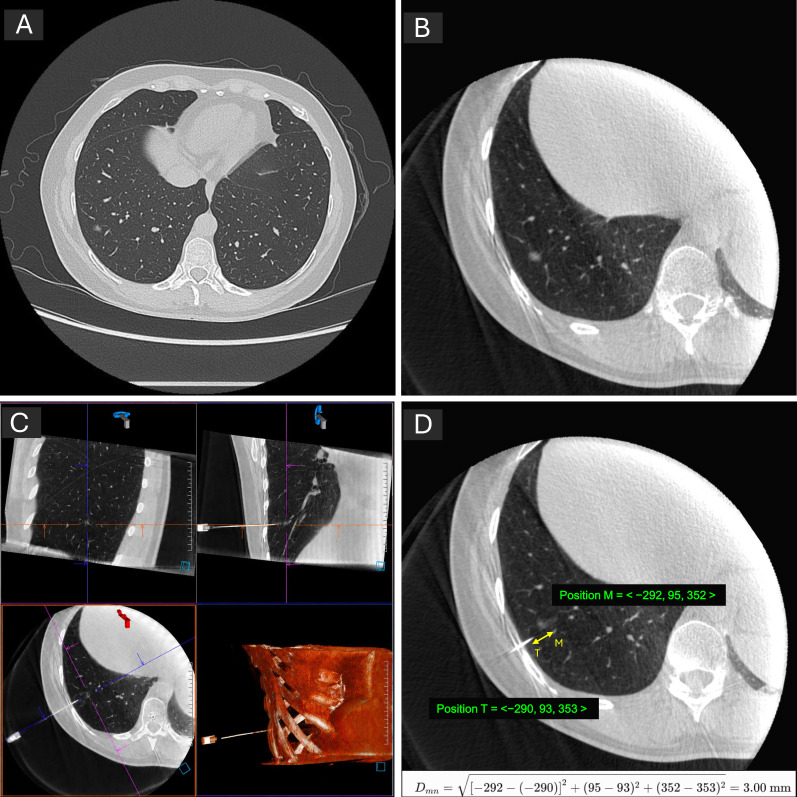
Representative imaging workflow for cone-beam CT-guided pulmonary nodule localization. (**A**) Preoperative thin-section CT showing a peripheral ground-glass nodule. (**B**) Intraoperative CBCT showing re-identification of the target lesion before needle placement. (**C**) Multiplanar and three-dimensional needle-guidance views used for target selection and trajectory planning. (**D**) Post-localization CBCT demonstrating the coordinate-based calculation of Dmn. T denotes the three-dimensional coordinate of the localization needle tip (−290, 93, 353), and M denotes the nearest point on the tumor boundary (−292, 95, 352). Dmn was calculated as the three-dimensional Euclidean distance between T and M and was 3.00 mm in this representative case.

Patients were placed in the lateral decubitus position or at an approximately 30° lateral tilt according to lesion location, and CBCT acquisition and localization were performed during end-inspiratory apnea. The localization method was not randomly assigned; hook-wire placement or Patent Blue V dye injection (Guerbet, Villepinte, France) was selected on a case-by-case basis, primarily according to the lesion-to-pleura distance. For hook-wire deployment, a 20-gauge Chiba needle (Hakko Co., Ltd., Chikuma, Nagano, Japan) was used as the introducer needle. For dye localization, following CBCT confirmation of the needle-tip position, 0.2 mL of Patent Blue V was injected through the localization needle using a 1 mL syringe near the target lesion or the anticipated resection plane. Immediately after localization, thoracoscopic wedge resection was performed in the same HOR. Representative preoperative CT, intraoperative CBCT, needle-trajectory planning, and post-localization images are provided in Figure 2.

All procedures were performed using VATS. Resected specimens were submitted for intraoperative frozen-section analysis. When adenocarcinoma in situ or minimally invasive adenocarcinoma was diagnosed, wedge resection was generally considered sufficient without additional anatomical resection, provided that the intraoperative and pathological findings were clinically acceptable. When invasive adenocarcinoma or another malignancy was confirmed, tumor size, pathological margin distance, lesion location, and technical feasibility were reviewed intraoperatively to determine whether additional wedge resection, segmentectomy, or lobectomy was required.

### 2.3. Pathological and Localization Assessment

For consistency, pathological margins were evaluated on the initial wedge specimen in all patients, regardless of frozen-section diagnosis or whether additional resection was subsequently performed. Margin distance was defined as the microscopic histologic distance between the tumor boundary and the resection edge, in accordance with current recommendations [24,25].

Localization error was quantified using Dmn, defined as the minimum Euclidean distance between the three-dimensional coordinate of the localization needle tip and the three-dimensional tumor boundary. Specifically, if $N=\left(x_{n},y_{n},z_{n}\right)$ denotes the needle-tip coordinate and $M_{i}=\left(x_{i},y_{i},z_{i}\right)$ denotes a point on the tumor margin, Dmn was calculated as $Dmn={min}_{i}\sqrt{{\left(x_{n}-x_{i}\right)}^{2}+{\left(y_{n}-y_{i}\right)}^{2}+{\left(z_{n}-z_{i}\right)}^{2}}$. A schematic representation of this measurement is provided in the Graphical Abstract. Patients were classified as having an adequate margin (MA; margin ≥ tumor size) or an inadequate margin (MI; margin < tumor size).

### 2.4. Statistical Analysis

Between-group comparisons were performed using the χ^2^ test, Fisher’s exact test, or Mann–Whitney U test, as appropriate. Logistic regression was used to identify predictors of MI, with Dmn specified as the primary covariate. Clinically relevant variables, including age, body mass index (BMI), contralateral lung surgery, localization method, lesion size, and lesion depth, were entered into the multivariable model. Backward stepwise selection was then performed to determine which variables were retained in the final model.

ROC analysis was performed to evaluate the predictive performance of Dmn, and the optimal cutoff was identified using Youden’s index. Cutoff stability was assessed using 1000 bootstrap resamples. In each resample, the optimal Dmn cutoff was recalculated using Youden’s index.

As Dmn did not follow a normal distribution, kernel density estimation (KDE) with highest-density region (HDR) analysis was applied to characterize the distribution of localization errors [28,29]. Spherical error probable (SEP), adapted from circular error probability analysis and spatial reliability assessment, was then calculated at the 50% (SEP50) and 95% (SEP95) levels to summarize the three-dimensional localization-error distribution [30,31,32]. A cumulative margin adequacy analysis was used to illustrate threshold-dependent changes in MA as a function of Dmn.

Supplementary sensitivity analyses were conducted in the combined cohort of central and peripheral lesions to assess whether including central lesions altered the primary findings. Exploratory surgeon-specific ROC analyses were also performed in the peripheral-lesion cohort, and pairwise comparisons of surgeon-specific areas under the curve (AUCs) were conducted using DeLong’s test for independent ROC curves.

All statistical analyses were performed using IBM SPSS Statistics (version 25; IBM Corp., Armonk, NY, USA), MATLAB (R2023a; MathWorks, Natick, MA, USA), and Microsoft Excel (Microsoft Corp., Redmond, WA, USA). A two-sided *p* value < 0.05 was considered statistically significant.

## 3. Results

### 3.1. Patient Characteristics

Overall, 169 patients with peripheral pulmonary lesions were included in the predefined primary analysis cohort and stratified according to pathological margin status. Of these, 138 patients (81.7%) were classified into the MA group and 31 (18.3%) into the MI group. Among patients with pathological margin inadequacy on the initial wedge specimen, two with invasive adenocarcinoma underwent conversion to lobectomy, whereas selected patients underwent additional wedge resection to obtain a wider gross surgical margin. Baseline demographic and clinical characteristics were comparable between the MA and MI groups, including age, sex, pulmonary function parameters, tumor size, lesion depth, procedure time, pneumothorax, lesion location, localization tool, history of contralateral lung surgery, and pathological subtype (Table 1).

Patients in the MI group had significantly higher Dmn values than did those in the MA group (median, 8.1 mm vs. 3.0 mm, *p* < 0.001) and smaller pathological margins (median, 5.0 mm vs. 11.0 mm, *p* < 0.001).

### 3.2. Backward Stepwise Logistic Regression Analysis

**Figure 3:**
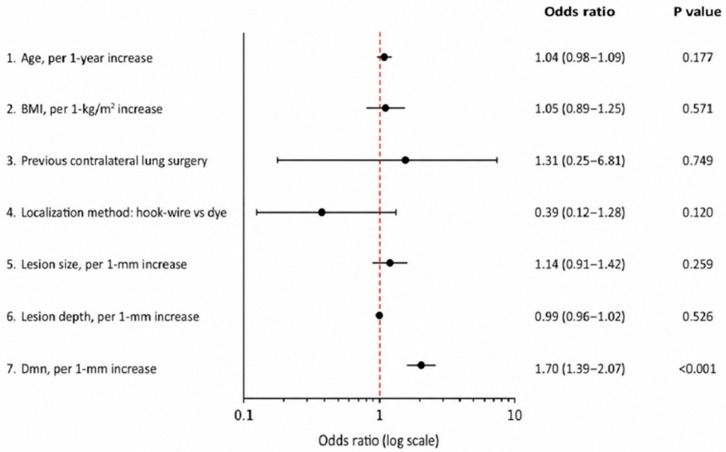
Multivariable logistic regression analysis for pathological margin inadequacy. Forest plot showing adjusted odds ratios (ORs) and 95% confidence intervals (CIs) from the initial multivariable logistic regression model. Dots represent adjusted ORs, horizontal lines represent 95% CIs, and the vertical dashed line at OR = 1 indicates no association. ORs are displayed on a logarithmic scale. Covariates included age, body mass index (BMI), previous contralateral lung surgery, localization method, lesion size, lesion depth, and Dmn. Dmn was defined as the shortest three-dimensional Euclidean distance from the localization needle tip to the tumor margin and was the only statistically significant independent predictor. BMI, body mass index; CI, confidence interval; OR, odds ratio.

In the initial multivariable logistic regression model, Dmn was the only statistically significant predictor of pathological margin inadequacy (Figure 3). After backward stepwise selection, Dmn remained the sole variable retained in the final model and was independently associated with pathological margin inadequacy (odds ratio [OR], 1.617; 95% confidence interval [CI], 1.356–1.928; *p* < 0.001) (Table 2). Each 1 mm increase in Dmn was associated with a 61.7% increase in the odds of pathological margin inadequacy.

### 3.3. ROC-Derived Cutoff and Clinical Risk Stratification

**Figure 4:**
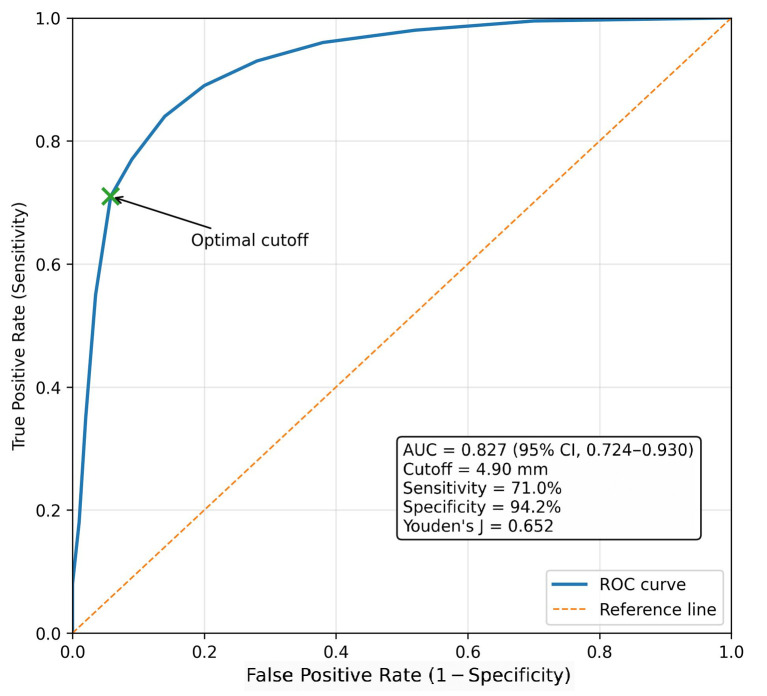
Receiver operating characteristic curve for Dmn in predicting pathological margin inadequacy. ROC analysis was performed using Dmn as the predictor and pathological margin inadequacy as the binary outcome. The optimal cutoff was determined by maximizing Youden’s index (sensitivity + specificity − 1). The ROC-derived cutoff was 4.90 mm, with an AUC of 0.827 (95% CI, 0.724–0.930), sensitivity of 71.0%, specificity of 94.2%, and Youden’s J of 0.652. AUC = area under the curve; ROC = receiver operating characteristic; Dmn = distance from the localization needle tip to the tumor margin.

ROC analysis yielded an AUC of 0.827, indicating good discriminatory performance of Dmn for predicting pathological margin inadequacy (Figure 4). Based on Youden’s index, the optimal Dmn cutoff was 4.90 mm, yielding a sensitivity of 71.0%, a specificity of 94.2%, and a Youden’s J of 0.652.

When stratified by the ROC-derived cutoff, patients with Dmn ≥ 4.9 mm had a markedly higher rate of pathological margin inadequacy than did those with Dmn < 4.9 mm (73.3% vs. 6.5%, *p* < 0.001) (Table 3). The crude OR for pathological margin inadequacy was 39.72 (95% CI, 13.84–113.98, *p* < 0.001), and the association remained significant after adjustment for lesion size, BMI, localization method, and previous contralateral lung surgery (adjusted OR, 50.09, 95% CI, 15.32–163.79, *p* < 0.001). At the 4.9 mm threshold, the positive predictive value was 73.3%, the negative predictive value was 93.5%, and the overall accuracy was 89.9%.

Across 1000 bootstrap resamples, the median bootstrap-derived cutoff was 4.90 mm (interquartile range [IQR], 4.90–5.10 mm; 2.5th–97.5th percentile, 4.58–5.83 mm), and the bootstrap median AUC was 0.830 (IQR, 0.795–0.861) (Supplementary Table S1 and Supplementary Figure S1).

### 3.4. Cumulative Margin Adequacy Analysis According to Dmn

**Figure 5:**
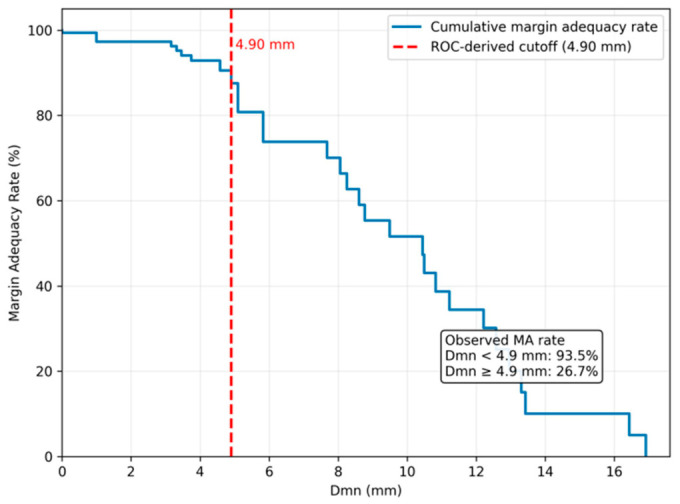
Cumulative margin adequacy rate according to Dmn. The blue step curve represents the cumulative pathological margin adequacy rate across increasing Dmn values. The red dashed vertical line indicates the receiver operating characteristic (ROC)-derived cutoff of 4.90 mm. When patients were stratified according to this cutoff, the observed margin adequacy rate was 93.5% in patients with Dmn < 4.9 mm and 26.7% in those with Dmn ≥ 4.9 mm. Dmn, distance from the localization needle tip to the tumor margin; MA, margin adequacy; ROC, receiver operating characteristic.

The cumulative margin adequacy curve demonstrated a progressive decline in pathological margin adequacy with increasing Dmn (Figure 5). Margin adequacy remained high at lower Dmn values but decreased markedly around the ROC-derived 4.9 mm cutoff.

### 3.5. Distribution Analysis of Localization Error

**Figure 6:**
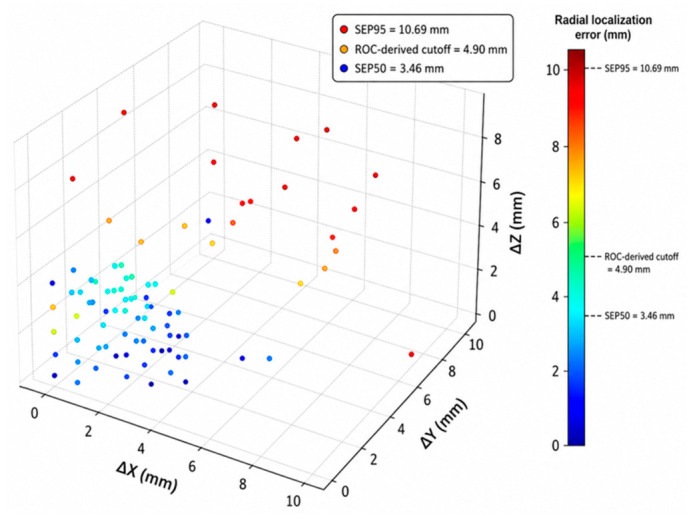
Three-dimensional distribution of localization-error vectors. Each point represents one localization attempt and is positioned according to the localization-error components ΔX, ΔY, and ΔZ. Point color represents the magnitude of radial localization error in millimeters, with larger values indicating greater localization error. Most localization attempts clustered near the target reference region, whereas a smaller number of high-error observations were more widely dispersed. SEP50, the ROC-derived cutoff, and SEP95 were 3.46 mm, 4.90 mm, and 10.69 mm, respectively, and are shown as reference values for interpreting the three-dimensional error distribution. ROC, receiver operating characteristic; SEP50, spherical error probable at the 50% level; SEP95, spherical error probable at the 95% level.

As Dmn did not follow a normal distribution, KDE with HDR analysis was applied to characterize the spatial distribution of localization error. Three-dimensional visualization demonstrated that most localization-error vectors clustered near the target reference region, whereas a smaller subset of high-error observations was more broadly dispersed (Figure 6).

**Figure 7:**
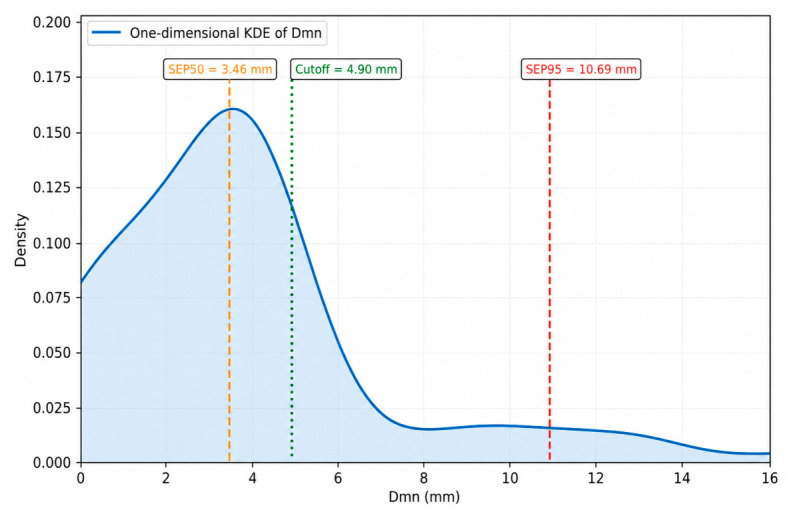
One-dimensional kernel density distribution of Dmn with SEP50, ROC-derived cutoff, and SEP95. Kernel density estimate of Dmn in the predefined peripheral-lesion cohort. The horizontal axis represents Dmn in millimeters, and the vertical axis represents estimated density. Vertical reference lines indicate SEP50 (3.46 mm), the ROC-derived cutoff (4.90 mm), and SEP95 (10.69 mm). The 4.9 mm cutoff lay within the upper quartile of the observed three-dimensional targeting-error distribution and closely approximated the 5 mm localization tolerance used in our clinical practice. Dmn, distance from the localization needle tip to the tumor margin; KDE, kernel density estimation; ROC, receiver operating characteristic; SEP50, spherical error probable at the 50% level; SEP95, spherical error probable at the 95% level.

The SEP values derived from the KDE-based distribution showed that 50% of cases lay within a 3.46 mm radius (SEP50), whereas 95% lay within 10.69 mm (SEP95) (Figure 7). The ROC-derived 4.9 mm cutoff lay within the upper quartile of the observed three-dimensional targeting-error distribution and closely approximated the 5 mm localization tolerance used in our clinical practice.

### 3.6. Supplementary Sensitivity Analysis Including Central Lesions

A supplementary sensitivity analysis was performed in the combined cohort of central and peripheral lesions to evaluate whether including central lesions altered the primary findings. The aim was to capture broader real-world heterogeneity, not to redefine the primary target population, because the prespecified primary analysis was restricted to peripheral lesions as the clinically more homogeneous wedge-resection cohort. In the combined cohort, ROC analysis showed an AUC of 0.778 for Dmn in predicting pathological margin inadequacy (Supplementary Figure S2). Using the same 4.9 mm cutoff, patients with Dmn ≥ 4.9 mm had a higher rate of pathological margin inadequacy than did those with Dmn < 4.9 mm (69.4% vs. 10.2%, *p* < 0.001) (Supplementary Table S2). The cutoff retained clinically meaningful performance: 64.2% sensitivity, 91.8% specificity, 69.4% positive predictive value, and 89.8% negative predictive value.

### 3.7. Exploratory Surgeon-Specific Analysis

Exploratory, surgeon-specific analyses were conducted in the predefined peripheral-lesion cohort to evaluate potential operator-related variability. Surgeon characteristics are summarized in Supplementary Table S4. Dmn and pathological margin distance varied across surgeons, whereas the rate of pathological margin inadequacy did not differ significantly.

Surgeon-specific ROC analysis showed numerical differences in AUCs among surgeons; however, pairwise DeLong comparisons did not demonstrate statistically significant between-surgeon differences in AUCs (Supplementary Table S5 and Supplementary Figure S3). Sample size and event counts varied across surgeon subgroups.

## 4. Discussion

In this study, Dmn was identified as the principal independent predictor of pathological margin inadequacy in the predefined peripheral-lesion cohort, thereby linking intraoperative localization accuracy with pathological margin adequacy. This finding is clinically relevant because inadequate resection margins have consistently been associated with an increased risk of local recurrence. Mohiuddin et al. [26] demonstrated that a greater margin distance significantly reduced local recurrence after wedge resection for small (≤2 cm) non-small cell lung cancer. Similarly, Wolf et al. [27] reported that larger margins were independently associated with lower recurrence and improved survival after wedge resection for stage I lung cancer. Collectively, these findings support the clinical relevance of pathological margin adequacy and provide a rationale for evaluating localization precision as a determinant of surgical margin safety.

Accordingly, our findings extend previous observations by demonstrating a direct relationship between three-dimensional localization accuracy and pathological margin adequacy, thereby linking a technical localization metric to an oncologically relevant surgical endpoint. ROC analysis identified a Dmn of 4.9 mm as the optimal cutoff for predicting pathological margin inadequacy, and internal bootstrap validation supported the stability of this threshold. In the cumulative margin adequacy analysis, adequacy declined markedly once Dmn exceeded 4.9 mm. Furthermore, KDE/HDR and SEP analyses placed the ROC-derived 4.9 mm cutoff within the upper quartile of the observed targeting-error distribution [28,29,30,31,32]. The convergence of ROC discrimination, bootstrap stability, cumulative margin adequacy analysis, and spatial-error distribution suggests that the 4.9 mm threshold reflects a genuine spatial characteristic of localization performance within this cohort rather than a statistical artifact. Similar three-dimensional error analyses have been applied in radiotherapy, navigation systems, stereotactic radiosurgery, and neurosurgery to quantify spatial targeting accuracy and safety margins [33,34,35,36,37].

To assess the robustness of the primary findings, we performed a supplementary sensitivity analysis that included central lesions (Supplementary Figure S2 and Supplementary Tables S2 and S3). Central lesions were excluded from the predefined primary analysis because wedge resection for centrally located nodules represents a more controversial and less standardized clinical scenario. In such cases, pathological margin adequacy may be influenced not only by localization accuracy but also by proximity to segmental bronchovascular structures, limitations in stapling geometry, and the potential need for anatomical resection. Although inclusion of central lesions modestly attenuated the discriminatory performance of Dmn, Dmn remained independently associated with pathological margin inadequacy, and the 4.9 mm threshold retained clinically meaningful performance. Nevertheless, extrapolation of this threshold to central lesions should be approached cautiously.

With the increasing detection of small and subsolid pulmonary lesions, accurate localization has become increasingly important for achieving adequate margins during parenchyma-sparing surgery. From a clinical perspective, until prospective external validation is available, the 4.9 mm value should complement, rather than replace, clinical judgment based on tumor size, lesion location, pathological findings, and the technical feasibility of achieving an adequate margin. Lower thresholds would increase sensitivity at the expense of more false-positive alerts and potentially unnecessary additional localization procedures, whereas higher thresholds would reduce sensitivity and increase the risk of missing inadequate margins. The ROC-derived 4.9 mm threshold closely approximated the 5 mm localization tolerance used in our clinical practice. In this cohort, 30 of 169 localization procedures (17.8%) had Dmn ≥ 4.9 mm and may warrant additional intraoperative margin reassessment. These findings also provide quantitative support for long-standing surgical principles, such as maintaining resection margins at least equivalent to tumor size and limiting localization error to approximately 5 mm.

Most previous studies have reported localization error in millimeters without directly correlating it with pathological margin adequacy, thereby limiting its oncologic interpretation. For example, a recent study using a three-dimensional printed localization device reported a mean localization error of 5.4 ± 4.2 mm. However, nearly half of the patients underwent segmentectomy, which complicated the interpretation of margin adequacy after wedge resection [38]. Similarly, Zhang et al. [39] reported a mean deviation of 9.6 ± 5.8 mm and a mean resection margin of 17.6 mm using template-assisted localization; however, more than one-quarter of patients ultimately underwent lobectomy. The inclusion of multiple surgical strategies may obscure the relationship between localization accuracy and pathological margin adequacy because larger anatomical resections can compensate for localization error. By restricting the primary analysis to peripheral lesions treated with initial wedge resection, the present study minimized confounding from anatomical resections and allowed a more direct evaluation of the relationship between three-dimensional targeting error and pathological margin adequacy.

When Dmn exceeds 4.9 mm, additional imaging, refinement of resection planning, or intraoperative reassessment of the margin may be considered. However, additional wedge resection should not be regarded as a reliable substitute for accurate initial localization and appropriate primary resection planning. After specimen removal, lung deflation and stapling-related deformation may make the true residual margin difficult to identify. Moreover, any additional localization or resection attempt must be balanced against the procedural risks associated with general anesthesia and positive-pressure ventilation, particularly pneumothorax requiring pleural drainage [40,41]. This issue may be especially relevant during hook-wire localization, because pneumothorax can destabilize the wire and increase the risk of dislodgement. Although no patient in this cohort underwent re-localization, future prospective studies are needed to determine whether threshold-guided intraoperative decision-making can safely improve pathological margin adequacy.

This study has several limitations. First, its retrospective, nonrandomized design means that selection bias cannot be excluded. Second, all procedures were performed at a single, high-volume institution by experienced thoracic surgeons. Although this setting minimized technical variability, it may limit the generalizability of the findings. Exploratory surgeon-specific analyses did not show statistically significant differences in AUCs among surgeons; however, these analyses were constrained by small subgroup sample sizes and event counts and should therefore be interpreted as supportive rather than definitive. Furthermore, because of the limited number of margin-inadequate events, multivariable modeling was restricted to clinically relevant variables, and the final model should be interpreted cautiously.

Third, the primary analysis was predefined for peripheral lesions. Although a supplementary sensitivity analysis including central lesions showed that Dmn remained independently associated with pathological margin inadequacy and that the 4.9 mm threshold retained clinically meaningful performance, discriminatory ability was modestly attenuated after central lesions were included. Therefore, extrapolation of this threshold to central lesions should be approached cautiously.

Fourth, the 4.9 mm cutoff was derived from a single-center cohort using a specific cone-beam CT system, image-acquisition and reconstruction protocol, localization workflow, respiratory management strategy, and experienced surgical team. Although bootstrap resampling supported the internal stability of this threshold, internal validation cannot substitute for external validation. Differences in imaging systems, tumor-margin segmentation, needle-tip identification, localization techniques, respiratory conditions, operator experience, and institutional surgical practices may affect Dmn measurement and its association with pathological margin adequacy. Moreover, although margin adequacy was defined relative to tumor size rather than by a fixed absolute margin, a single Dmn threshold may not have identical clinical implications across different tumor sizes or histological subtypes. The present cohort predominantly comprised GGN-spectrum adenocarcinomas, and the limited number of margin-inadequate events precluded the reliable derivation of tumor-size-specific or histology-specific thresholds. Therefore, the 4.9 mm value should be interpreted as a center-derived candidate intraoperative warning threshold rather than a definitive, universally applicable decision rule. Prospective external validation across independent institutions, imaging systems, localization techniques, and surgeons is required before routine clinical implementation. Individual centers should also confirm the reproducibility and local performance of Dmn measurement, with center-specific recalibration considered if systematic differences are identified.

Fifth, pathological margin adequacy of the initial wedge specimen served as the primary endpoint. This endpoint was selected because it directly reflects the adequacy of the first intended wedge resection after localization. Accordingly, MI in this study should be interpreted as pathological margin inadequacy of the initial wedge specimen rather than final surgical treatment failure. Although additional resection was considered when clinically indicated, the feasibility and reliability of additional wedge resection may be limited by intraoperative anatomical constraints and uncertainty in identifying the true residual margin. Moreover, this study was not designed to evaluate long-term oncological outcomes. Therefore, the prognostic significance of the 4.9 mm threshold requires further validation using longer follow-up and recurrence-based endpoints.

Sixth, pathological margin distance was defined as the microscopic distance between the tumor boundary and the resection edge on the initial wedge specimen. Although this definition is consistent with current recommendations, other studies may use alternative measurement approaches, including gross specimen measurements or pleural-based assessments, which may limit cross-study comparability.

Finally, these findings are most applicable to solitary GGN-spectrum pulmonary nodules managed using cone-beam CT-guided localization and thoracoscopic wedge resection in a hybrid operating room. Their applicability to multiple nodules, segmentectomy, lobectomy, or other localization platforms remains uncertain.

## 5. Conclusions

Dmn was the principal independent predictor of pathological margin inadequacy in the predefined peripheral-lesion cohort. A 4.9 mm cutoff demonstrated good discriminatory performance and identified markedly different rates of margin inadequacy in patients with Dmn ≥ 4.9 mm and <4.9 mm, while bootstrap and sensitivity analyses supported the internal robustness of this association. A Dmn of approximately 4.9 mm may therefore serve as a candidate intraoperative warning value for margin reassessment and localization-quality evaluation; however, prospective external validation is required before routine clinical implementation.

## Figures and Tables

**Table 1:** Baseline characteristics of included patients.

Variable	Inadequate Pathological Margin (MI)	Adequate Pathological Margin (MA)	*p* Value
	*n* = 31	*n* = 138	
Age, years, median (IQR)	55.0 (46.0–62.0)	50.0 (44.0–57.0)	0.161
Sex, *n* (%)			0.256
Female	26 (83.9)	101 (73.2)	
Male	5 (16.1)	37 (26.8)	
FVC, %, median (IQR)	101.0 (90.0–111.5)	103.0 (93.0–113.0)	0.561
FEV1, %, median (IQR)	98.0 (84.0–107.5)	100.5 (91.0–110.0)	0.343
DLCO, %, median (IQR)	91.0 (84.0–100.0)	94.0 (85.0–104.0)	0.352
Tumor size, mm, median (IQR)	8.5 (6.80–10.50)	7.5 (6.30–9.00)	0.054
Depth, mm, median (IQR)	65.0 (55.0–75.0)	60.0 (45.0–75.0)	0.128
Error distance (Dmn), mm, median (IQR)	8.1 (4.2–11.0)	3.0 (1.4–4.1)	<0.001
Pathological margin, mm, median (IQR)	5.0 (3.0–6.0)	11.0 (9.0–13.0)	<0.001
Procedure time, min, median (IQR)	11.0 (9.0–14.0)	11.0 (8.0–14.0)	0.746
Pneumothorax, *n* (%)	3 (9.7)	8 (5.8)	0.423
Lesion location, *n* (%)			0.460
LLL	3 (9.7)	28 (20.3)	
LUL	7 (22.6)	36 (26.1)	
RLL	3 (9.7)	17 (12.3)	
RML	1 (3.2)	10 (7.2)	
RUL	17 (54.8)	47 (34.1)	
Localization tool, *n* (%)			0.686
Dye	18 (58.1)	86 (62.3)	
Hook-wire	13 (41.9)	52 (37.7)	
Contralateral lung operation, *n* (%)			0.740
No contralateral lung operation	28 (90.3)	126 (91.3)	
With a previous contralateral lung operation	3 (9.7)	12 (8.7)	
Pathology, *n* (%)			0.408
Adenocarcinoma in situ	9 (29.0)	49 (35.5)	
Invasive adenocarcinoma	6 (19.4)	15 (10.9)	
Minimally invasive adenocarcinoma	16 (51.6)	74 (53.6)	

Baseline demographics, tumor characteristics, localization parameters, and peri-procedural outcomes stratified by surgical margin adequacy. Continuous variables are presented as median (interquartile range, IQR); categorical variables are presented as numbers (%). Dmn = distance from the localization needle tip to the tumor margin; FVC = forced vital capacity; FEV1 = forced expiratory volume in 1 s; DLCO = diffusion capacity of carbon monoxide; LLL = left lower lobe; LUL = left upper lobe; RLL = right lower lobe; RML = right middle lobe; RUL = right upper lobe. Categorical variables were compared using the χ^2^ test or Fisher’s exact test; continuous variables were analyzed using the Mann–Whitney U test.

**Table 2:** Multivariable backward stepwise logistic regression for pathological margin inadequacy.

Group	Variable	OR	95% CI	*p*
Primary peripheral cohort	Dmn	1.617	1.356–1.928	<0.001

Values are presented as odds ratio, 95% confidence intervals, and *p* values. Candidate variables entered the initial model included age, BMI, prior contralateral lung surgery, localization method, lesion size, lesion depth, and Dmn. Backward stepwise selection was performed, and only variables retained in the final model are shown. Dmn represents the shortest Euclidean distance from the localization needle tip to the tumor margin, based on three-dimensional localization coordinates. CI = confidence interval; OR = odds ratio.

**Table 3:** Clinical performance of the ROC-derived 4.9 mm Dmn cutoff for predicting pathological margin inadequacy.

Variable	Dmn < 4.9 mm	Dmn ≥ 4.9 mm	*p* Value
Patients, *n*	139	30	—
Margin adequate, *n* (%)	130 (93.5%)	8 (26.7%)	<0.001
Margin inadequate, *n* (%)	9 (6.5%)	22 (73.3%)	<0.001
Diagnostic performance	Value		
Sensitivity	71.0%		
Specificity	94.2%		
Positive predictive value	73.3%		
Negative predictive value	93.5%		
Accuracy	89.9%		
Positive likelihood ratio	12.24		
Negative likelihood ratio	0.31		
Crude odds ratio	39.72 (95% CI, 13.84–113.98)		
Adjusted odds ratio	50.09 (95% CI, 15.32–163.79)		

Dmn was defined as the shortest Euclidean distance from the localization needle tip to the tumor margin. Margin inadequacy was defined as a pathological margin smaller than the maximum tumor diameter. The adjusted odds ratio was calculated after adjustment for lesion size, BMI, localization method, and contralateral lung surgery. CI = confidence interval.

## Data Availability

The data supporting the findings of this study are not publicly accessible due to institutional regulations and patient privacy considerations. De-identified data may be provided by the corresponding author upon reasonable request.

## Supplementary Materials

The following supporting information can be downloaded at https://www.mdpi.com/article/10.3390/cancers18142356/s1, Figure S1: Bootstrap distribution of optimal Dmn cutoffs; Figure S2: Sensitivity ROC analysis in the combined central and peripheral-lesion cohort; Table S1: Internal bootstrap validation of the ROC-derived Dmn cutoff; Table S2: Clinical performance of the 4.9 mm Dmn cutoff in the combined central and peripheral-lesion cohort: A. Risk stratification according to the 4.9 mm Dmn cutoff. B. Diagnostic performance of the 4.9 mm Dmn cutoff; Table S3: Sensitivity backward stepwise multivariable logistic regression in the combined central and peripheral-lesion cohort; Table S4: Surgeon-specific characteristics in the peripheral-lesion cohort; Table S5: Surgeon-specific ROC analysis and pairwise DeLong comparison in the peripheral-lesion cohort: A. Surgeon-specific ROC analysis. B. Pairwise DeLong comparison of surgeon-specific AUCs; Figure S3: Surgeon-specific ROC curves in the peripheral-lesion cohort.

## Abbreviations

AUC	area under the curve
BMI	body mass index
CBCT	cone-beam computed tomography
CI	confidence interval
CT	computed tomography
Dmn	distance from the localization needle tip to the tumor margin
GGN	ground-glass nodule
HDR	highest-density region
HOR	hybrid operating room
IQR	interquartile range
KDE	kernel density estimation
MA	margin adequacy
MI	margin inadequacy
OR	odds ratio
ROC	receiver operating characteristic
SEP	spherical error probable
SEP50	spherical error probable at the 50% level
SEP95	spherical error probable at the 95% level
TALENT	Taiwan Lung Cancer Screening in Never-Smoker
VATS	video-assisted thoracoscopic surgery
