# Supplementary material for: Bridging Targeting Precision and Oncologic Safety: Localization Accuracy for Margin Adequacy in Cone-Beam Computed Tomography-Guided Pulmonary Nodule Resection
Source: Cancers (Basel). 2026 Jul 21;18(14):2356. doi: 10.3390/cancers18142356 (PMC13406559; doi:10.3390/cancers18142356)
Supplement: Supplementary file 1 [file cancers-18-02356-s001.zip › cancers-4437383-supplementary.pdf]

Supplementary Materials

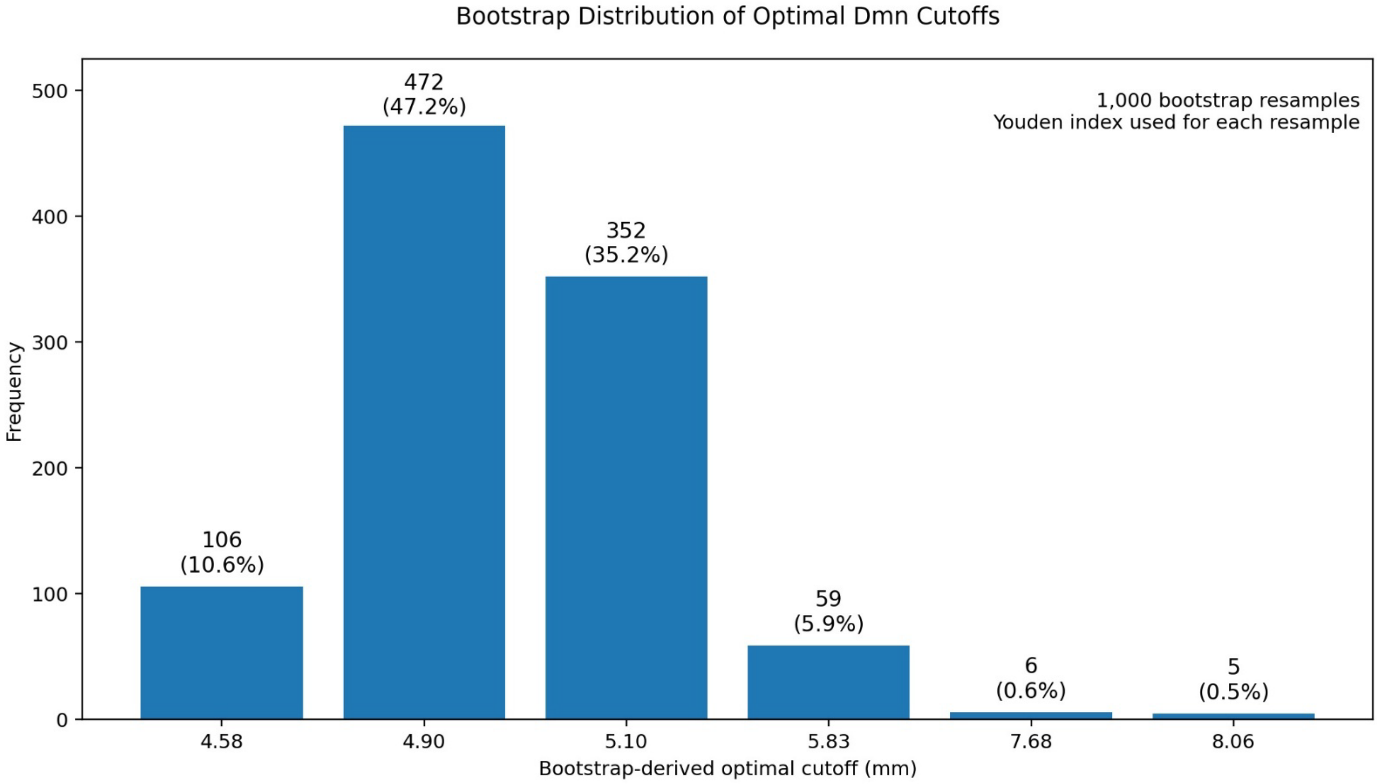

Supplementary Figure S1. Bootstrap distribution of optimal Dmn cutoffs.
Optimal Dmn cutoffs were recalculated in 1,000 bootstrap resamples using the Youden index. Most bootstrap-derived optimal cutoffs clustered around 4.90 and 5.10 mm, supporting the internal stability of the ROC-derived 4.9-mm candidate threshold for Dmn, defined as the distance from the localization needle tip to the tumor margin.

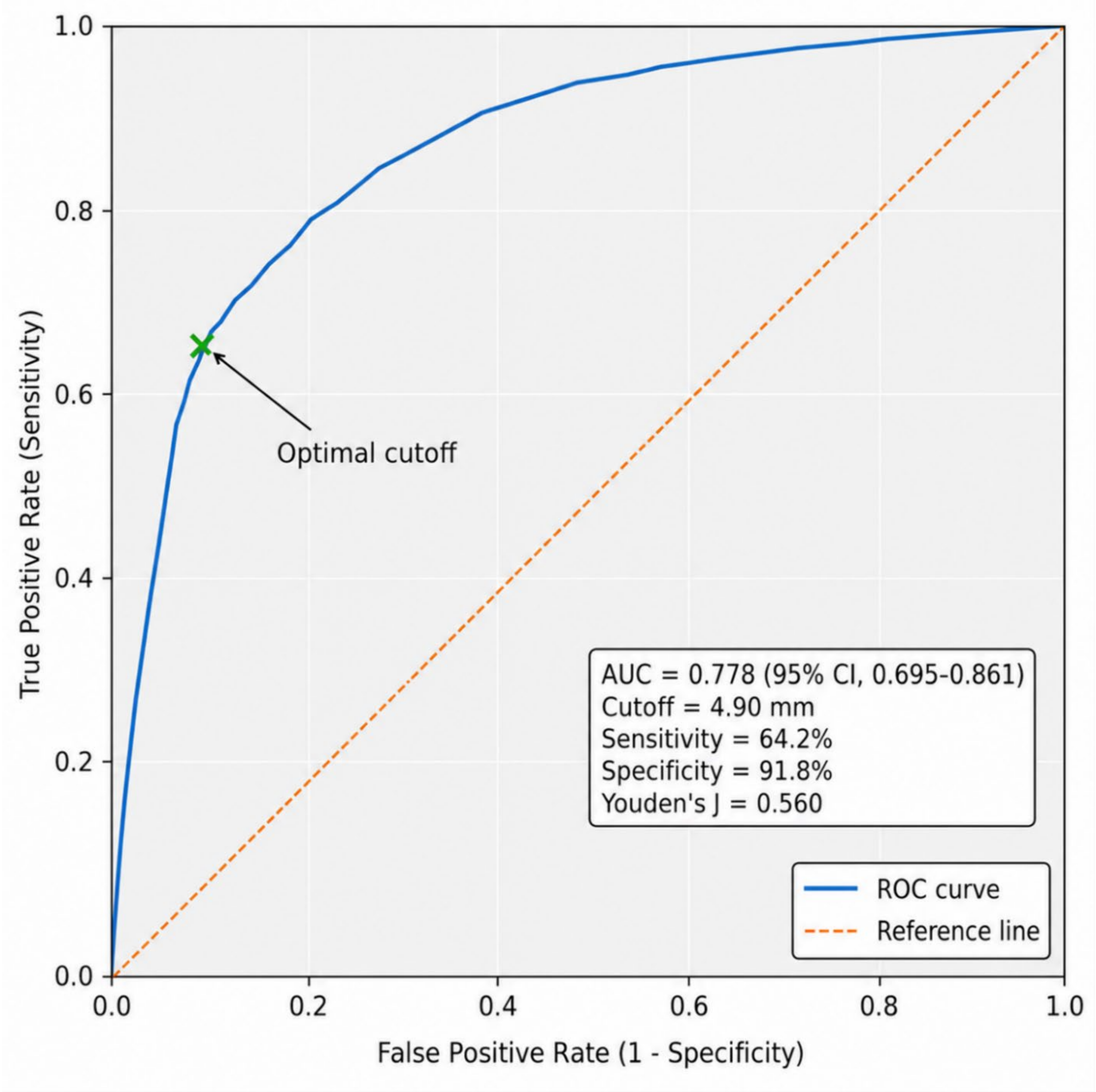

Supplementary Figure S2. Sensitivity ROC analysis in the combined central and peripheral lesion cohort. Receiver operating characteristic (ROC) analysis was repeated in the combined central and peripheral lesion cohort to assess whether inclusion of central lesions altered the primary conclusion derived from the predefined peripheral-lesion cohort. The AUC was 0.778, and independent optimization using Youden's index again identified an optimal Dmn cutoff of 4.90 mm, identical to that derived from the predefined peripheral-lesion cohort. At this cutoff, sensitivity was 64.2% and specificity was 91.8%. These findings indicate that inclusion of central lesions attenuated but did not materially change the main conclusion that greater Dmn is associated with pathological margin inadequacy. Dmn, distance from the localization needle tip to the tumor margin.

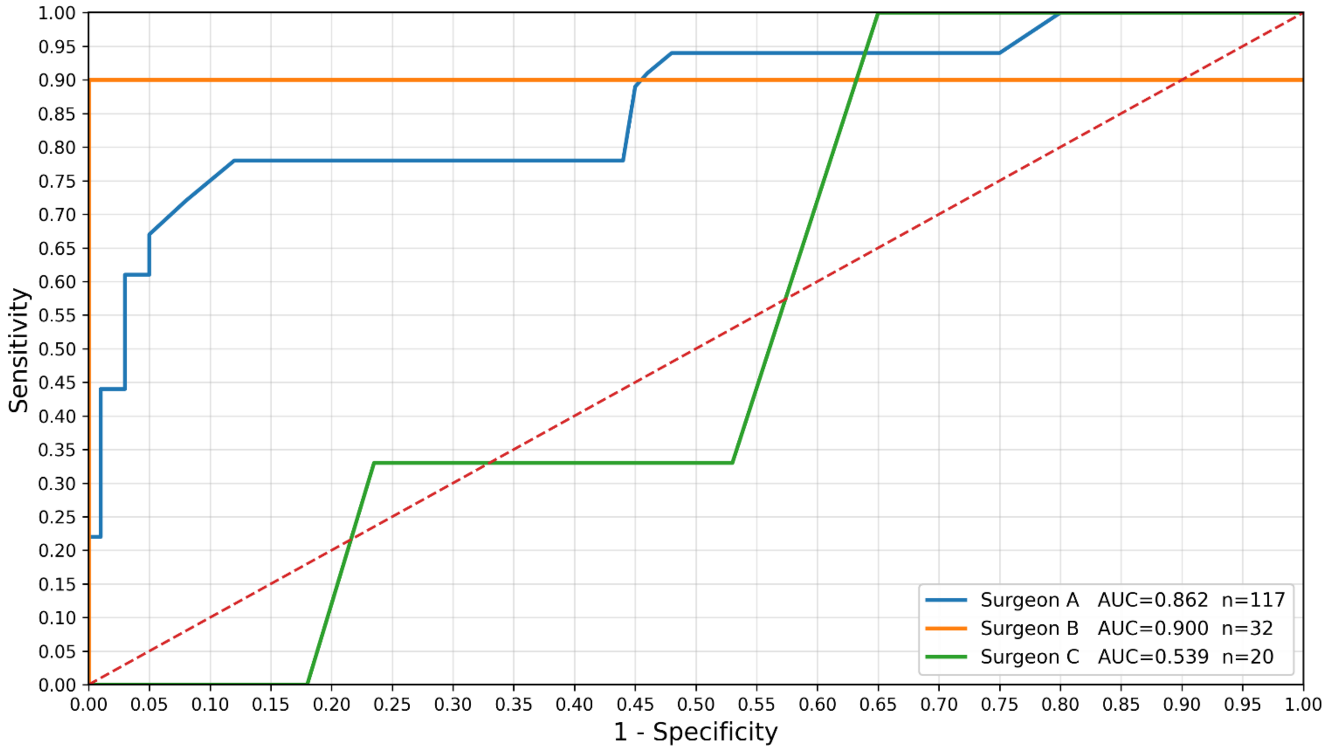

Supplementary Figure S3. Surgeon-specific ROC curves in the peripheral-lesion cohort. Receiver operating characteristic (ROC) curves of Dmn for predicting pathological margin inadequacy are shown separately for Surgeons A, B, and C in the predefined peripheral-lesion cohort. The area under the curve (AUC) was 0.862 for Surgeon A, 0.900 for Surgeon B, and 0.539 for Surgeon C. Pairwise comparisons of AUCs using DeLong's test for independent ROC curves did not reveal statistically significant differences among surgeons (A vs B, $p = 0.742$; A vs C, $p = 0.056$; B vs C, $p = 0.056$). Owing to the limited sample size and event count in the smaller surgeon subgroups, these analyses should be interpreted as exploratory. Dmn, distance from the localization needle tip to the tumor margin.

Supplementary Table S1. Internal bootstrap validation of the ROC-derived Dmn cutoff

Metric	Original estimate	Bootstrap median	IQR	2.5th–97.5th percentile
AUC	0.827	0.830	0.795–0.861	0.722–0.925
Cutoff (mm)	4.899	4.899	4.899–5.099	4.583–5.831
Sensitivity	0.710	0.714	0.667–0.769	0.545–0.871
Specificity	0.942	0.955	0.933–0.970	0.861–0.992

ROC, receiver operating characteristic; Dmn, distance from the localization needle tip to the tumor margin; AUC, area under the receiver operating characteristic curve; IQR, interquartile range.

Supplementary Table S2. Performance of the 4.9-mm Dmn cutoff in the predefined peripheral-lesion cohort and the combined cohort of central and peripheral lesions

A. Risk stratification according to the 4.9-mm Dmn cutoff

Cohort	Margin status	Dmn < 4.9 mm	Dmn ≥ 4.9 mm	p value
Peripheral cohort	Patients, n	139	30	—
	Margin adequate, n (%)	130 (93.5%)	8 (26.7%)	<0.001
	Margin inadequate, n (%)	9 (6.5%)	22 (73.3%)	
Combined central + peripheral cohort	Patients, n	186	49	—
	Margin adequate, n (%)	167 (89.8%)	15 (30.6%)	<0.001
	Margin inadequate, n (%)	19 (10.2%)	34 (69.4%)	

B. Diagnostic performance of the 4.9-mm Dmn cutoff

Metric<br>Patients, n	Peripheral cohort	Combined central + peripheral cohort
	N = 169	N = 235
MI events, n	31	53
AUC	0.827	0.778
Cutoff, mm	4.90	4.90
Sensitivity	71.0%	64.2%
Specificity	94.2%	91.8%
Positive predictive value	73.3%	69.4%
Negative predictive value	93.5%	89.8%
Accuracy	89.9%	85.5%
Positive likelihood ratio	12.24	7.78
Negative likelihood ratio	0.31	0.39
Crude odds ratio	39.72 (95% CI, 13.84–113.98)	19.92 (95% CI, 9.22–43.07)
Adjusted odds ratio	50.09 (95% CI, 15.32–163.79)	23.93 (95% CI, 9.93–57.64)

The peripheral-lesion cohort constituted the predefined primary analysis cohort, whereas the combined cohort of central and peripheral lesions was evaluated as a supplementary sensitivity analysis. Dmn was defined as the shortest three-dimensional Euclidean distance from the localization needle tip to the tumor margin. Margin inadequacy was defined as a pathological margin smaller than the maximum tumor diameter. In both cohorts, the adjusted odds ratio was estimated after adjustment for lesion size, body mass index, localization method, and prior contralateral lung surgery. AUC, area under the receiver operating characteristic curve; BMI, body mass index; CI, confidence interval; Dmn, distance from the localization needle tip to the tumor margin; OR, odds ratio.

Supplementary Table S3. Sensitivity analysis using backward stepwise multivariable logistic regression in the combined central and peripheral lesion cohort.

Variable	OR	95% CI	p
Lesion size	1.282	1.087–1.512	0.003
Dmn	1.551	1.343–1.792	<0.001

Backward stepwise logistic regression was performed in the combined central and peripheral lesion cohort as a supplementary sensitivity analysis. Candidate variables entered into the initial model included age, BMI, prior contralateral lung surgery, localization method, lesion size, lesion depth, and Dmn. Only variables retained in the final model are shown. Dmn remained independently associated with pathological margin inadequacy after inclusion of central lesions. CI, confidence interval; Dmn, distance from the localization needle tip to the tumor margin; OR, odds ratio.

Supplementary Table S4. Surgeon-specific characteristics in the peripheral-lesion cohort

Variable	Surgeon A	Surgeon B	Surgeon C	p value
n	117	32	20	
MI, n (%)	18 (15.4%)	10 (31.3%)	3 (15.0%)	0.111
Depth, mm, median [IQR]	60 [45–70]	62.5 [55–80]	55 [40–76.25]	0.209
Lesion size, mm, median [IQR]	8.0 [6.5–9.6]	7.5 [5.88–8.03]	7.1 [6.5–8.4]	0.031
Error distance, mm, median [IQR]	3.46 [1.41–4.36]	4.12 [3.00–5.28]	1.50 [0–3.19]	0.001
Pathological margin distance, mm, median [IQR]	10 [8–13]	8 [5–10.25]	10 [7–13.5]	0.011

IQR, interquartile range; MI, margin inadequacy.

Supplementary Table S5. Surgeon-specific ROC analysis and pairwise DeLong comparison in the peripheral-lesion cohort.

A. Surgeon-specific ROC analysis

Surgeon	n	MI events	AUC	95% CI
Surgeon A	117	18	0.862	0.753–0.972
Surgeon B	32	10	0.900	0.704–1.000
Surgeon C	20	3	0.539	0.226–0.852
All patients	169	31	0.827	0.724–0.930

B. Pairwise DeLong comparison of surgeon-specific AUCs

Comparison	AUC difference	z statistic	p value
Surgeon A vs Surgeon B	−0.038	−0.330	0.742
Surgeon A vs Surgeon C	0.323	1.908	0.056
Surgeon B vs Surgeon C	0.361	1.914	0.056

ROC analysis was performed separately for each surgeon using Dmn as the predictor and pathological margin inadequacy as the outcome. Pairwise comparisons of surgeon-specific AUCs were performed using DeLong's test for independent ROC curves. These analyses were exploratory and should be interpreted cautiously because of the limited sample size and event count in the smaller surgeon subgroups. Dmn, distance from the localization needle tip to the tumor margin; AUC, area under the receiver operating characteristic curve; CI, confidence interval; ROC, receiver operating characteristic.
